# Enhanced Photodynamic Therapy: A Review of Combined Energy Sources

**DOI:** 10.3390/cells11243995

**Published:** 2022-12-10

**Authors:** José A. Rodrigues, José H. Correia

**Affiliations:** 1 CMEMS-UMinho, University of Minho, 4800-058 Guimarães, Portugal; 2 LABBELS-Associate Laboratory, 4800-122 Braga, Portugal

**Keywords:** photodynamic therapy, non-visible radiation, ultrasound, electric field, magnetic field, synergistic strategies

## Abstract

Photodynamic therapy (PDT) has been used in recent years as a non-invasive treatment for cancer, due to the side effects of traditional treatments such as surgery, radiotherapy, and chemotherapy. This therapeutic technique requires a photosensitizer, light energy, and oxygen to produce reactive oxygen species (ROS) which mediate cellular toxicity. PDT is a useful non-invasive therapy for cancer treatment, but it has some limitations that need to be overcome, such as low-light-penetration depths, non-targeting photosensitizers, and tumor hypoxia. This review focuses on the latest innovative strategies based on the synergistic use of other energy sources, such as non-visible radiation of the electromagnetic spectrum (microwaves, infrared, and X-rays), ultrasound, and electric/magnetic fields, to overcome PDT limitations and enhance the therapeutic effect of PDT. The main principles, mechanisms, and crucial elements of PDT are also addressed.

## 1. Introduction

### 1.1. Principles of Photodynamic Therapy and Photodynamic Reaction

Photodynamic therapy (PDT) is a therapeutic modality that is based on the combination of three factors to promote the selective destruction of a target tissue: photosensitizer (PS), light with a specific wavelength, and the presence of molecular oxygen [1,2,3,4]. None of them are toxic individually, but together they initiate a photochemical reaction that culminates in the generation of reactive oxygen species (ROS) responsible for oxidative cell damage that can lead to the destruction of the target tissue [5,6,7,8]. The typical PDT procedure consists of two sequential steps: administration (intravenous or topically) of a PS and subsequent irradiation using light of a specific wavelength (usually a red lamp or laser beam) at the tumor’s location. Between PS administration and light irradiation, an appropriate time interval (drug–light interval) is required for the photosensitizer to accumulate in the tumor [5,9]. The drug–light interval depends on the route of administration, the type of PS, and its pharmacokinetic and biodistribution properties [10].

**Figure 1:**
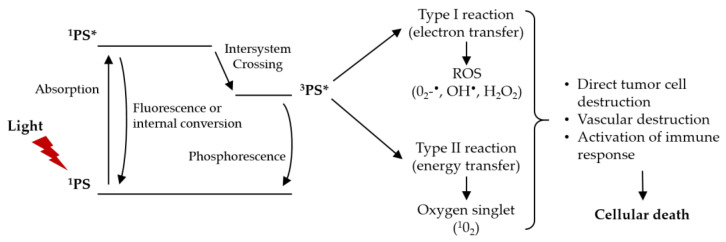
Modified Jablonski diagram of the PDT action mechanism. Adapted from [7].

The photodynamic reaction starts with the absorption of light by the PS in the target tissue, which triggers a series of photochemical reactions that lead to the generation of ROS [7,9,10,11,12]. The PS in its ground state (singlet state, ^1^PS) has a stable electronic configuration, i.e., it has two electrons with opposite spins. The absorption of a photon of light of a specific wavelength leads the PS to a short-lived (a few nanoseconds) electronically excited singlet state (^1^PS*). This excited state is very unstable, losing the excess of energy by emitting light (fluorescence) or producing heat (internal conversion). However, the singlet state can undergo a process known as intersystem crossing to form a more stable, long-lived (10^−6^ to 10^−3^ s), electronically excited state (triplet state, ^3^PS*). The PS in the triplet state can decay back to the ground state through the emission of light (phosphorescence) or undergo two types of reactions. The long lifetime of the triplet state is sufficient to transfer its energy directly to the molecular oxygen (O_2_). This energy transfer step leads to the formation of singlet oxygen (^1^O_2_) and the ground state of a PS, called a type II reaction [7,9,11,13,14]. The type I reaction can also occur if the PS in the excited state reacts directly with a cellular substrate, such as the cell membrane or a molecule, and undergoes electron transfer reactions, forming ROS. This mechanism may involve the acquisition or donation of an electron to form cationic or anionic radicals, respectively. These radicals react with molecular oxygen, producing ROS, such as superoxide anion radical (O^2−•^), hydroxyl radical (HO^•^), and hydrogen peroxide (H_2_O_2_) [7,9,11,13]. Figure 1 shows the modified Jablonski diagram of the PDT action mechanism.

The products resulting from type I and II reactions lead to tumor ablation by three interrelated mechanisms: direct cytotoxic effects on tumor cells (apoptosis and necrosis), indirect damage to the tumor-associated vasculature, and induction of an inflammatory response that can activate an immune response against the tumor cells [8,9,11,12,13]. Both reactions can occur simultaneously, however the ratio between these two processes depends on the type of PS used, concentrations of substrate and molecular oxygen, as well as the binding affinity of PS to the substrate. Due to the high reactivity and short half-life of the ROS, only cells that are close to the ROS production area (PS location area) are directly affected by PDT [7,14]. The extent of damage and cytotoxicity resulting from PDT is multifactorial, depending on the type of PS, its extracellular and intracellular location and the total dose administered, light dose (light fluence) and light fluence rate, availability of oxygen, and drug–light interval [5,7,10,12].

### 1.2. Light, Photosensitizers and Oxygen

Light is an essential component of PDT. The clinical efficacy of PDT is highly dependent on the accuracy of light delivery to the target tissue and its dose, which translates into light fluence rate, light fluence, light exposure time, and light delivery mode (single or fractionated) [7,15]. Light-fluence rate is the incident energy per second across a sectional area of the irradiated spot and is expressed as W/cm^2^. Light fluence is the total energy of exposed light across a sectional area of an irradiated spot and is expressed in J/cm^2^ [7]. These two parameters define the light exposure time, which is obtained by dividing the light fluence by the light fluence rate. High light doses in a short period of time, i.e., high light fluence rates, have been reported as a non-ideal practice in PDT, reducing its efficiency. The main reason for this is the rapid tissue oxygen depletion, limiting the generation of reactive oxygen species and thus the potential for tumor destruction. Furthermore, low light-fluence rates increase the selective apoptosis of tumor cells, which is more desirable than the inflammation and edema that usually occurs with the uncontrolled rupturing of cellular contents during necrosis [7,12,15].

Molecular oxygen is a fundamental element for PDT-induced cytotoxicity. So, tissue oxygenation is extremely important for the efficacy of PDT. The oxygen concentration can vary significantly between different tumors and even between different regions of the same tumor, depending on the density of the vasculature. Especially in deeper solid tumors, often characterized by their anoxic microenvironment, lack of oxygen can be a limiting factor. As mentioned above, the light fluence rate is related to photochemical oxygen depletion during the generation of cytotoxic singlet oxygen by PDT. Oxygen depletion occurs when the oxygen consumption rate by the photodynamic reaction is greater than the rate of oxygen diffusion in the irradiated area. Continuous adjustment of the light fluence rate (maintaining the total light dose) during PDT allows the oxygen consumption rate to not exceed the rate of oxygen diffusion into the target tissue. This balance can also be achieved by using fractionated light [7,10].

Another essential component of PDT, besides light and oxygen, is the presence of PSs. These substances are defined as substances capable of absorbing light with a specific wavelength and triggering photochemical or photophysical reactions [1,16]. Some of the features that should be found in an ideal PS are [5,7,10,12,16,17,18,19]:

- High purity and chemical stability;
- Strong absorption with a high molar extinction coefficient (ε) for higher light wavelengths (600 to 800 nm);
- High singlet oxygen quantum yield (Φ_Δ_);
- Low dark toxicity;
- Accumulation and retention, preferentially in the target tissues and rapid clearance from healthy tissues;
- Amphiphilicity;
- Inexpensive, simple synthesis and easy availability.

Most of the PSs used in PDT are porphyrins or their reduced derivatives, such as chlorins or bacteriochlorins, which have in common the tetrapyrrole macrocycle, similar to that of the protoporphyrin contained in hemoglobin [5,10]. The first compounds to demonstrate therapeutic potential for PDT of cancer were hematoporphyrin derivatives (HpD), of which the purified version and commercially approved porfimer sodium (Photofrin^®^) represents the first-generation of PS [1,5,10,16]. PDT for cutaneous indications commonly utilizes a topical photosensitizer, such as 5-aminolevulinic acid or methyl aminolevulinate, which are precursors of protoporphyrin IX. Treatment of visceral tumors requires an intravenous or oral photosensitizer, and the most commonly used photosensitizing agent for this indication is porfimer sodium [15]. The second-generation PSs arose to overcome some drawbacks of the first-generation ones. The second-generation PSs are characterized by a higher chemical purity, higher yield of singlet oxygen formation, and better light penetration to deeply located tissues, due to their maximum absorption in the wavelength range 650–800 nm. The third-generation PSs are molecules with improved selectivity for tumor regions, due to the conjunction of the PS with targeting molecules (antibody conjugates) or its encapsulation into carriers (e.g., liposomes micelles, nanoparticles) [12,15,16]. Table 1 shows some PS used in the PDT.

### 1.3. Limitations of Photodynamic Therapy

Like other therapies, the breadth and depth of PDT’s effectiveness have not been fully realized due to limitations, such as depth of light penetration, inefficient PSs, targeted delivery, and tumor hypoxia [2,11,17]. When considering PDT as a treatment option, the accuracy of target tissue irradiation is the most important point because PDT is effective only when light hits the target area. So, deep tumors (not easily accessible without surgical intervention) are difficult to treat due to the low penetration of visible light into the tissue.

Disseminated metastases are also very difficult to treat with the currently available technology [2,7,17]. The difficulty in systemic administration of this form of therapy is because PSs are generally easy to aggregate and lack targeting, limiting the clinical efficacy of PDT [2,7,11,17]. Furthermore, due to the excessive proliferation of cancer cells and insufficient blood supply in the tumors, the O_2_ content in the tumors is severely insufficient, resulting in a significant reduction in the effectiveness of PDT [7,8,11,17].

**Figure 2:**
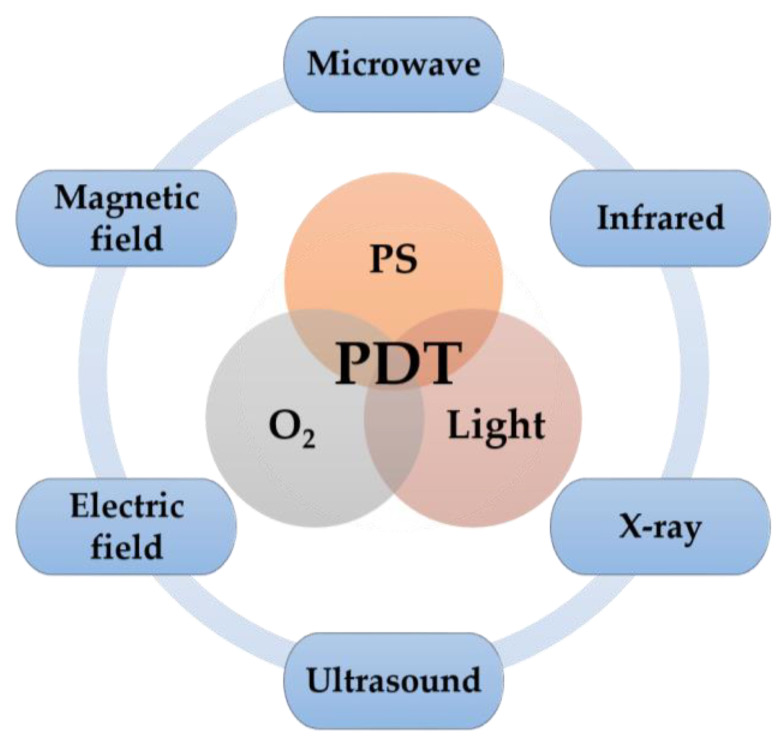
Schematic illustration of other energy sources (in addition to the visible light source) that can be used in PDT to overcome its limitations and enhance its therapeutic effect.

In recent years, great efforts have been devoted to overcoming the limitations of PDT and a number of strategies have been proposed to increase its efficiency [11]. In this review, we focus on the latest innovative strategies based on the use of non-visible radiation of the electromagnetic spectrum (microwaves, radio waves, infrared, and X-rays), ultrasound, and electric/magnetic fields to overcome PDT limitations and enhance its therapeutic effect (Figure 2).

## 2. Illumination of the Tissue

Different types of illumination sources have been proposed for photodynamic treatment. The choice of the light source should be based on the PS absorption spectrum, pathology characteristics (location, lesion size, accessibility, and tissue characteristics), and cost. Typically, tissue illumination in PDT can be performed by four different types of light sources: lamps, light-emitting diodes (LEDs), lasers, and daylight [5,7,10,19,25].

### 2.1. Lamp Light

Lamps have the advantage of being affordable, portable, easy to use, requiring low maintenance, having the ability to cover a large area, and providing a wide spectral output. This type of light source includes halogen, xenon, and metal-halide lamps. The use of narrowband filters allows the selection of a range of wavelengths that correspond to the maximum absorption of the PS. Optical filtering is also required to ensure unnecessary ultraviolet and infrared wavelengths are removed from the output light. Loss of energy in the form of heat, low-light intensity, and restriction of irradiation to easily accessible places (e.g., skin) are some disadvantages of this type of light source [2,7,10,26].

### 2.2. Light-Emitting Diodes

LEDs are characterized by fixed narrowband emission compared with lamps, eliminating the need for optical filters. They can be assembled to cover large areas of irradiation or complex anatomic shapes. LEDs have advantages over other PDT light sources, such as low cost and low hazard. In addition, LEDs have other advantages: they are compact, lightweight, thermally non-destructive, easily available in flexible arrays, and require low amounts of energy when producing desired wavelengths [2,5,10,19,27].

### 2.3. Laser Light

The development of the laser was a major milestone in PDT and is currently the most widely used light source. There are four types of laser light sources that have been used in PDT: argon-pumped lasers, metal-vapor-pumped lasers (Au- or Cu-vapor lasers), solid-state lasers (Nd:YAG lasers, Ho:YAG lasers, KTP:YAG/dye lasers), and diode lasers. Lasers produce high-intensity coherent monochromatic light. They can be coupled to optical fibers to reach inaccessible locations (e.g., lung and bladder) and decrease light loss due to scattering. For the treatment of superficial lesions, in order to cover a relatively large target tissue with uniform irradiance, the laser can be coupled with beam-expanding lenses. Irradiation through lasers with a defined wavelength facilitates the accurate calculation of the light dose. The complex, bulky, and expensive laser systems used in the past have been replaced by easy-to-use, reliable, and cost-effective laser diodes [5,10,19,25,26,28].

### 2.4. Daylight

Another relevant light source for PDT is natural light. The concept of daylight PDT is based on the use of natural light instead of an artificial light source to treat skin lesions, such as actinic keratosis. Daylight PDT has made PDT more widespread, cheaper, less painful, and with shorter clinic visits (patients can complete their therapy at home) [2,7]. However, important drawbacks include the difficulty of scheduling due to daylight dependence on weather and times, as well as the inconvenience of controlling daylight exposure [2].

### 2.5. Light Absorption in Biological Tissues

**Figure 3:**
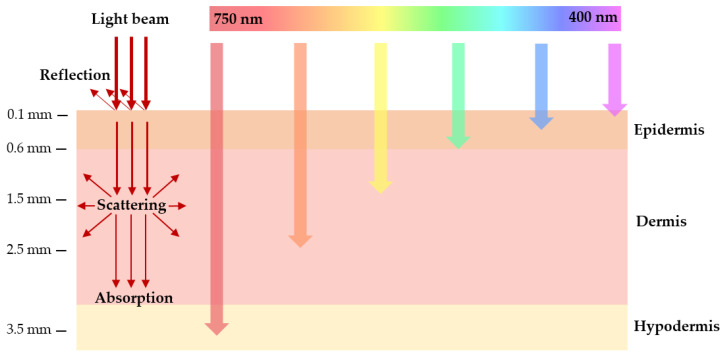
Schematic representation of wavelength-dependent light penetration into biological tissue. The schematic shows a section of the skin layers (epidermis, dermis, and subcutaneous layer). The arrows represent the penetration power of light with different wavelengths in the tissues. Blue light penetrates less efficiently into the tissue, while red light penetrates more deeply.

Light penetration into biological tissue is very complex, as it can be reflected, scattered, or absorbed. The extent of these processes depends on the type of tissue and light wavelength (Figure 3) [7,10,18,29]. Light absorption is mainly due to endogenous chromophores existing in tissues, such as hemoglobin, myoglobin, melanin, and cytochromes, which can decrease the photodynamic process by competing with PS in the absorption process. The region between 600 and 1200 nm is often called the “tissue optical window”, due to the absorption of lower wavelength light by the endogenous chromophores, combined with reduced light scattering at longer wavelengths and the occurrence of water absorption at wavelengths greater than 1200 nm [5,7,18,30]. Shorter wavelengths (<600 nm) have less tissue penetration and are more absorbed, resulting in high skin photosensitivity. On the other hand, longer wavelengths (>850 nm) do not have enough energy to generate triplet states of PS that can efficiently transfer their energy to molecular oxygen. Therefore, the highest tissue permeability occurs between 600 and 850 nm. This band, called the “phototherapeutic window”, is predominantly used in PDT [7,10,18].

## 3. Enhanced PDT

The typical PDT procedure is based on the irradiation of tumor tissue with visible light. In recent years, new strategies have been used to overcome the limitations of PDT and enhance its therapeutic effect. These strategies rely on the use of non-visible radiation of the electromagnetic spectrum, ultrasound, and electric/magnetic fields. 

### 3.1. Non-Visible Radiation

**Figure 4:**
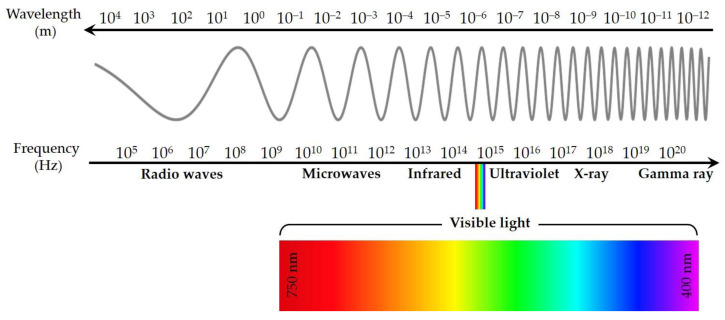
Electromagnetic spectrum.

Visible light corresponds to a narrow slice of the electromagnetic spectrum that is visible to the human eye. However, electromagnetic radiation also includes non-visible radiation, such as radio waves, microwaves, infrared, ultraviolet, X-ray, and gamma radiation (Figure 4) [6].

#### 3.1.1. Microwaves

Microwaves have been widely explored for tumor ablation in clinical settings due to their depth of penetration into tissues, high heating efficiency, and negligible side effects [31,32]. Microwave ablation uses electromagnetic waves in the microwave energy spectrum to produce tissue heating effects that ultimately generate tissue necrosis within solid tumors. Microwave energy is capable of propagating through all types of tissue and non-metallic materials, including water vapor and dehydrated, charred, and desiccated tissue created during the ablative process [33,34]. The combination of microwaves and PDT has been reported as a new therapy for deep cancer treatment. Microwave irradiation can cause local hyperthermia. When tissues are heated, blood vessels dilate and blood flow increases, thereby enhancing the effectiveness of PDT treatment [2,34]. Several PSs for microwave-induced PDT have been used to produce ROS under microwave irradiation and destroy tumor cells, such as copper-cysteamine (Cu–Cy) nanoparticles [33], g-C_3_N_4_ quantum dots [35], TiO_2_ nanoparticles [36], Fe-metal organic framework nanoparticles [37], liquid metal supernanoparticles [31], Cu_2_ZnSnS_4_ nanocrystals [38], Mn-doped zirconium metal-organic framework nanocubes [39], and gold nanoparticles [40]. 

#### 3.1.2. Infrared Light

The most direct approach to excite PS for PDT in deeper tissues is to use radiation that lies within the NIR (near-infrared) optical window (700 to 1100 nm) [41]. Compared to visible light, NIR light minimizes the degree of tissue scattering with a penetration depth greater than 1 cm. The use of NIR light not only allows deeper penetration of the tumor but also reduces phototoxicity in healthy tissues [11]. There are three main approaches for using NIR radiation to perform PDT in deeper tissues: two-photon absorption, nonlinear optical photon conversion techniques, and the use of upconversion materials and nanoparticles [29].

A single NIR photon does not have enough excitation energy to produce singlet oxygen molecules and induce photodynamic tissue damage [41]. Two-photon absorption is a nonlinear optical process involving the simultaneous absorption of two infrared photons that combined, promote an electron to a higher energy level than a single photon [29,42,43,44]. A key feature of two-photon excitation is the nonlinearity of photon absorption which makes it possible to activate PSs at the focal point of the laser beam. This allows for better spatial control of PS activation in three dimensions during PDT, reducing off-target damage to surrounding healthy tissues [29,41,44]. Several studies revealed that two-photon excitation of a commonly used PS was insufficient to induce PDT phototoxicity in vivo, so PSs specific for direct two-photon excitation were designed [29,41]. The first proof-of-concept in vivo study for two-photon PDT was reported in 2008 by H. A. Collins et al., who developed a new family of porphyrin-based PSs with high two-photon cross-sections [42,45]. Since then, other PSs were developed, as can be seen in [46,47,48,49,50,51]. An effective PS for two-photon PDT requires both the maximal two-photon absorption cross-section (e.g., gold nanorods) and a sufficient singlet oxygen quantum yield (e.g., porphyrin derivatives) [41]. To enable direct excitation of the PS through two-photon absorption, light sources with ultra-fast pulses of high photon density (femtosecond laser) are typically required due to the low absorption probability of the two-photon absorption process in most PSs [29,42].

PSs can also be excited indirectly (also using ultra-fast high-intensity lasers) by exploiting nonlinear optical photon conversion mechanisms occurring in many biological tissue constituents. Second-harmonic generation is a second-order nonlinear optical process that occurs in collagen (abundant in tumors). Four-wave mixing, including coherent anti-Stokes Raman scattering, is a third-order nonlinear optical process produced by the natural intracellular macromolecules (proteins and lipids). Kachynski et al. demonstrated deeper light penetration and phototoxicity effects with lower radiation thresholds by using a combination of these novel techniques as compared to two-photon absorption alone. With the same irradiation dose, 4500 J/cm^2^, the two-photon excitation was at the threshold level of phototoxicity, while these new techniques showed that 70% of the cells were necrotic or detached [29,52].

Another indirect excitation of the PSs is based on the upconversion of NIR photons into visible photons using dedicated upconversion materials, such as nano-transducers, or upconverting nanoparticles (UCNPs) [29]. There has been an increased interest in using energy nano-transducers to locally absorb incident NIR radiation to subsequently activate the PS. These nano-transducers can have various origins: chromophores [53,54], plasmonic gold nanorods [55,56], semiconductor quantum dots [57], and carbon quantum dots [58]. Regarding UCNPs, they are usually made of a ceramic lattice doped with rare-earth ions (lanthanides) that allow the sequential absorption of two photons through a metastable energy level [41,59]. The metastable state lifetime is typically on the order of a microsecond, which makes it possible to use continuous wave lasers and, more importantly, lower energy densities for UCNP excitation (1–10^3^ W/cm^2^ for UCNP excitation vs. 10^6^–10^9^ W/cm^2^ for two-photon activation) [41]. UCNPs have the ability to convert NIR light to visible light, which can then activate PS through the transfer of electronic excitation energy, either radiative (i.e., absorption of upconverting luminescence photons by PS) or non-radiative (i.e., via Förster resonance energy transfer or Dexter mechanisms of the electronic excitation energy transfer) [41,44,60]. Several strategies for conjugating UCNPs and PSs have been developed: PSs can be covalently conjugated to UCNPs through surface functionalization and chemical binding procedures, PSs can be non-covalently attached to the surface of UCNPs through either hydrophobic–hydrophobic interactions or electrostatic interactions, PSs can be embedded in a mesoporous silica matrix to coat the UCNPs, and a core-shell architecture comprising the UCNPs as the core and PS (TiO_2_, ZnO) as the shell [42,60,61]. There are a large number of in vitro and in vivo studies reporting an efficient effect of UCNPs-induced PDT, however their in vivo bioclearance and toxicity still need thorough investigation to reinforce clinical applicability [41].

#### 3.1.3. X-ray

**Figure 5:**
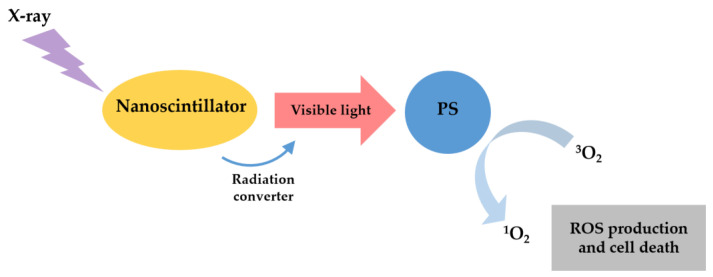
Schematic illustration of the classic X-PDT. X-rays excite a nanoscintillator to generate X-ray luminescence, which in turn activates a PS to produce cytotoxic ROS.

X-ray has been widely applied in clinical tumor imaging and therapy because X-ray photons have an unlimited penetration depth in the human body compared to visible or near-infrared light [2,11,32]. Recently, PDT and X-ray have been merged to establish a new mode of PDT to treat deep tumors, X-ray-induced PDT (X-PDT) [26,62]. Figure 5 shows the schematic illustration of the classic X-PDT. PS cannot be directly excited by X-rays because of the significant energy mismatch between therapeutic X-rays and PS [26]. So, the PS must be activated using radioluminescence of nanoparticles: scintillation nanoparticles (SCNPs) or persistent luminescence nanoparticles (PLNPs) [2,26,29,63].

SCNPs downconvert X-ray energy into visible light through a scintillation process and then transfer the energy to nearby PSs to initiate PDT [26,29,64]. SCNPs can be classified into two major groups: doped scintillators and semiconductors [29,64]. Some important characteristics are a high material density (for a good interaction with ionizing radiation), high scintillation quantum yield and efficient energy transfer, biocompatibility, and adapted in vivo biodistribution [29]. The doped scintillators based on lanthanides elements have been the most widely explored, due to their high material density, high atomic number, and strong luminescence intensity [29,64]. Table 2 shows some SCNPs used in X-PDT and the energy required to activate them.

PLNPs store X-ray energy at the defects or electron traps, causing a long-lasting afterglow that continuously emits light for a relatively long time (a few minutes to several days) for PDT activation [26,29]. This allows X-PDT to remain active in the absence of external irradiation, which can lead to reduced X-ray exposure for normal tissues [64]. Despite the promising results, most X-ray-induced PDT studies have been performed on cancer cell lines or animal models with subcutaneously grafted cancer cells, thus limiting the clinical relevance [29].

Cherenkov radiation has also been proposed to generate light and activate PDT in deep tissues using ionizing radiation [26,29,41,64]. Cherenkov emission is observed when charged particles, e.g., electrons or positrons, travel faster than the phase velocity of light in a given medium [29,32,41,77]. Research shows that Cherenkov radiation from medical radioisotopes can activate PSs to produce ROS [32,64]. Cherenkov-PDT has been reported using both radionuclides [78,79,80] and radiotherapy beams [81,82]. One strategy is to pair a radionuclide and a photosensitizer in a nanoparticle package [80]. Alternatively, a co-localization approach can be used, where radionuclides and photosensitizers are injected separately but co-enriched in cancer cells [64]. Radionuclide-induced Cherenkov radiation offers some potential advantages: suppression of external irradiation sources, minimization of healthy tissue exposure, and targeting multiple metastases with high selectivity (many radiopharmaceuticals are able to selectively accumulate in tumors after systemic injection). The main problem is the extremely low fluence rates of Cherenkov radiation [29,64]. Monte Carlo simulations have shown that fluence rates for radionuclides are in the order of 0.01–1 nW/cm^2^ per MBq/g. Such fluence rates are insufficient to produce effective phototoxicity [64]. Cherenkov-PDT can also be stimulated by external irradiation. This approach is attractive because, compared to radioisotopes, external beam-induced Cherenkov radiation may provide a higher photon flux and thus more efficient PDT [64].

### 3.2. Ultrasound

**Figure 6:**
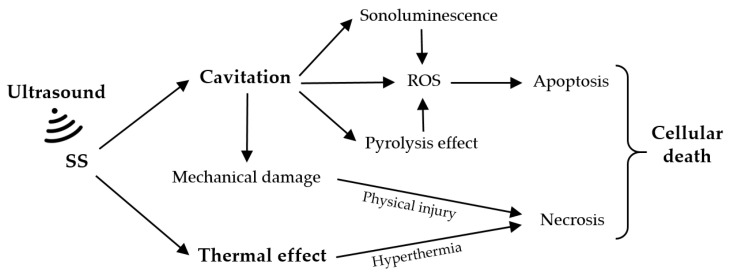
Possible SDT-mediated action mechanisms. Adapted from [87].

Ultrasound refers to sound frequencies that are above the range of human hearing (>20 kHz) [2,32,83]. Unlike visible light, ultrasound is a type of mechanical wave that can penetrate deeply into a tumor target [84]. Compared to PDT, sonodynamic therapy (SDT) is an analogous approach based on the synergistic effect of ultrasound and a chemical compound called a sonosensitizer (SS) [83,85]. Ultrasound can precisely focus on specific tumor sites and effectively activate the cytotoxicity of SSs, triggering the destruction of tumor cells with minimal damage to the adjacent normal tissues [11,83,84]. The SDT-mediated action mechanisms depend on biological models, the type of SSs, and ultrasound exposure parameters, including frequency and intensity [84,86]. However, it is difficult to define a universal mechanism of action. At present, the possible theories include the production of ROS, the cavitation effect, and thermal damage (Figure 6) [32,87,88,89,90].

The cavitation effect is caused by the interaction between the ultrasound and the aqueous environment, which involves the nucleation, growth, and implosion of gas bubbles under proper ultrasound irradiation [87,88,89]. Cavitation can be classified into two types, stable cavitation and inertial cavitation. The oscillation of stable cavitation bubbles results in the flow of the surrounding media. In contrast, inertial cavitation involves the growth of gas bubbles to resonance size and then to maximum size before wild collapse [91,92]. When bubbles rapidly collapse, inertial cavitation causes energy to be released, resulting in cell necrosis from high temperature and pressure [87]. Cavitation can lead to sonoluminescence and a pyrolysis effect. Sonoluminescence refers to the generation of light due to inertial or stable cavitation, which can further excite the SS to produce electrons and holes, and then ROS in the aqueous environment like PDT [89,91]. In the pyrolysis effect, the increase in local temperature, which accompanies the inertial cavitation process, decomposes the SS and generates free radicals via water pyrolysis, which then react with other endogenous substrates to produce ROS that induce tumor cell apoptosis [87,89,90,91]. Another ultrasound-mediated therapeutic mechanism is the thermal effect on tissues. The absorption and transformation of ultrasound mechanical energy induces the generation of thermal energy during the process of ultrasound wave propagation in tissues, leading to cell necrosis [87].

Organic porphyrins and their derivatives, including protoporphyrin [93,94], hematoporphyrin [95], and hematoporphyrin monomethyl ether [96,97,98] have been widely used as SS in SDT. However, most organic SSs face problems, such as strong hydrophobicity/poor water solubility, short circulation time in the physiological environment, and low tumor enrichment efficiency, which limit their further application [87,89,91]. To overcome these limitations, some inorganic-based SSs have been used in SDT, such as TiO_2_ [99,100], ZnO [101], Fe_3_O_4_ [102], MnWO*_x_* [103], Au [104], and black phosphorus [105,106]. Compared with organic SSs, inorganic SSs have better stability, low phototoxicity, and unique physiochemical properties, making them circulate more in the blood. The disadvantages of inorganic SSs are dose–dependent toxicity and low ultrasound absorption efficiency [87]. 

Recently, SDT and PDT have been combined to treat cancer synergistically, giving rise to sono-photodynamic therapy (SPDT). The basis of SPDT is the administration of a small amount of a SPDT sensitizer and its activation by ultrasound and/or light irradiation. The combination of SDT and PDT produces more ROS than both therapies alone, which can reduce the sensitizer dosage and enhance cytotoxicity [91,107,108]. So far, many SPDT sensitizers have been investigated, such as porphyrin derivatives [109,110], chlorin e6 [107,111], rose bengal [112], and sonnelux [113]. Many factors can affect the efficacy of SPDT: ultrasound and light parameters, tumor type, the physicochemical and biological properties of the sensitizers, and the sequence of SDT and PDT [107]. Wang et al. [114] indicated that SPDT was more effective in killing MDA-MB-231 cells (breast tumor cells) than PDT or SDT alone, with SDT before PDT more powerful than SDT after PDT. Similar results were obtained by Zhang et al. [115], Bakhshizadeh et al. [116], and Aksel et al. [117] using SPDT in the treatment of breast cancer (clinical trial), colorectal cancer, and prostate cancer.

### 3.3. Eletric/Magnetic Field

**Figure 7:**
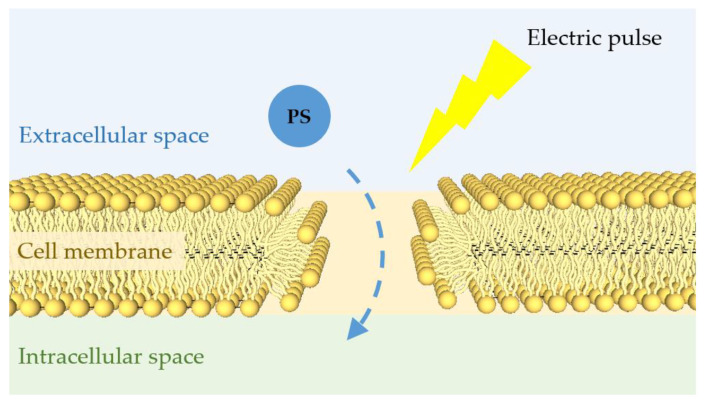
Schematic of the electroporation effect. A short external electric pulse of high voltage can affect the organization of the cell membrane generating non-selective pores. These pores allow the transport of PS into the cell and thus enhance the PDT effect.

An electric field of sufficiently high energy affects the cell membrane by generating non-selective pores (reversible electroporation) or destroying the membrane (irreversible electroporation) [2,11,118]. Figure 7 shows a schematic of the electroporation effect. The reversible electroporation supports the non-selective transport of non-permeant molecules into the cell, which can be used for gene transfection or drug delivery [2,118]. One of the most crucial elements of PDT is the ability of a PS to induce efficient transmembrane transport and intracellular accumulation [119]. Numerous studies have used the electroporation effect to help PS pass through the cell membrane [119,120,121,122,123]. The results demonstrated that electric pulses used in combination with PDT enhance photodynamic effectiveness. A major advantage of electroporation-based PDT is the greater selectivity of the treatment, since the PS transport rate is high only in the area of application of the electric field.

A magnetic field takes advantage of its intrinsic penetrability and harmlessness to the human body, making it a candidate for synergistic therapy with conventional PDT [124]. The non-directional accumulation of the PS in the tumor cells remains a major challenge for PDT, causing normal cells in the vicinity of the tumor to suffer the toxic effect from light activation [125]. To overcome this challenge, the magnetic field has been explored. The conjugation of PSs with biocompatible superparamagnetic nanomaterials enhances the directional accumulation of PSs into the tumor via their response to an external magnetic field [2,17,125]. Magnetic field-mediated PDT has the advantage of rapidly concentrating the magnetic nanomaterial and its load within the target environment, requiring a relatively lower dose to achieve therapeutic action [125]. The most common nanomaterials used in magnetic field-mediated PDT are functionalized superparamagnetic iron oxide nanoparticles (SPIONPs), such as Fe_3_O_4_ [126,127,128]. These nanomaterials have some advantages, such as relatively high biocompatibility, functional surfaces, low toxicity, and efficient superparamagnetism [125].

## 4. Conclusions

PDT is one of the most interesting and promising approaches to treat various oncologic diseases. This therapeutic technique requires a photosensitizer, light energy, and oxygen to produce ROS which mediate cellular toxicity. PDT is a useful non-invasive therapy for cancer treatment, however it has some limitations that need to be overcome, such as low-light-penetration depths, non-targeting photosensitizers, and tumor hypoxia. In this review, we described some strategies based on the synergetic use of different energy sources (including microwaves, infrared, X-rays, ultrasound, and electric/magnetic fields) to overcome the limitations of PDT and enhance its therapeutic effect. The main principles, mechanisms, and crucial elements of PDT were also addressed. Table 3 summarizes the contribution of different energy sources in overcoming the limitations of PDT.

There are two main criteria to facilitate the clinical translation of these strategies: therapeutic superiority and clinical safety. There is still a need to improve PDT strategies and conduct clinical studies to demonstrate its effectiveness compared to other treatment modalities such as surgery and chemotherapy. The nanoparticles/nanomaterials used in most of these strategies still need investigation in terms of in vivo bioclearance and toxicity to reinforce their clinical applicability. However, synergetic strategies focused on combining different energy sources continue to be crucial for pursuing and improving the effectiveness of PDT.

The final goal of this review is to promote future innovative studies that aim to overcome the limitations of PDT and reveal its full clinical potential.

## Figures and Tables

**Table 1:** Examples of PS used in the PDT [1,5,7,9,10,16,20,21,22,23,24].

Photosensitizer (Generation)	λ of Max. Absorption (nm)	Main Applications
Porfirmer sodium or Photofrin (1st generation)	630	FDA approved: treatment of carcinomas Phase I clinical trials: ovarian, breast, skin metastases Phase II clinical trials: lung, head and neck, bladder, brain Phase III clinical trials: esophagus, bile duct
Fimaporfin or Amphinex (2nd generation)	633	Phase I clinical trials: Superficial cancers, colon Phase II clinical trials: bile duct
5-aminolevulinic acid or Levulan (2nd generation)	635	FDA approved: skin Phase I/II clinical trials: bladder, brain, esophagus
Methyl-aminolevulinate or Metvix (2nd generation)	635	FDA approved: skin
Hexyl-aminolevulinate or Hexvix (2nd generation)	635	FDA approved: bladder Phase I/II clinical trials: prostate, colon
Temoporfin or Foscan (2nd generation)	652	EU approved: advanced head and neck squamous cell carcinoma Phase II clinical trials: lung, brain, bile duct, pancreas, skin, breast
Talaporfin (2nd generation)	660	Japan approved: early-stage endobronchial carcinoma Phase II clinical trials: brain, liver, colon, breast, skin metastases
Rostaporfin or Purlytin (2nd generation)	660	Phase II/III clinical trials: breast, bile duct, ovarian
Bremachlorin (2nd generation)	662	Phase II clinical trials: skin, lung
HPPH ^1^ or Photochlor (2nd generation)	665	Phase II clinical trials: head and neck, esophagus, lung
Ce6-PVP ^2^ or Photolon (2nd generation)	665	Phase II clinical trials: skin, lung, brain
Verteporfin or Visudyne (2nd generation)	690	FDA approved: choroidal neovascularization in wet age-related macular degeneration (AMD) Phase I/II clinical trials: pancreas, breast
Motexafin lutetium or Lutrin (2nd generation)	732	Phase I clinical trials: prostate, breast, ovarian, colon, stomach, skin metastases
Redaporfin or LUZ11 (2nd generation)	749	Phase II clinical trials: head and neck, biliary tract
Padeliporfin or Tookad (2nd generation)	762	EU approved: prostate Phase I/II clinical trials: esophagus

^1^ 2-(1-hexyloxyethyl)-2-devinyl pyropheophorbide-a; ^2^ chlorin e6-polyvinylpyrrolidone.

**Table 2:** Some SCNPs (nanoscintillators and photosensitizers) used in X-PDT. The energy required to activate them is also presented. Adapted from [29,64].

X-ray Scintillator (Emission)	Photosensitizer (Absorption)	X-ray Energetics	Ref
CeF_3_ (340 nm)	Verteporfin (370, 420 nm)	6 MeV, 30 keV, 1–6 Gy	[65]
SrAl_2_O_4_:Eu^2+^ (520 nm)	Merocyanine (540 nm)	50 keV, 1–10 Gy	[66]
LaF_3_:Ce^3+^ (520 nm)	PpIX ^1^ (409 nm)	90 keV, 3 Gy	[67]
LaF_3_:Tb (544 nm)	Rose Bengal (560 nm)	75 keV	[61]
LaF_3_:Tb silica coated (540 nm)	Rose Bengal (560 nm)	75 keV	[68]
LaF_3_:Tb (540 nm)	MTPC ^2^ (516 nm)	80 keV	[69]
GdEuC12 (595 nm)	Hypericin (590 nm)	15 keV	[70]
Hf-nMOL (500 nm)	Ir[bpy(ppy)_2_]^+^ (355 nm) or [Ru(bpy)_3_]^2+^ (450 nm)	225 keV, 2 Gy	[71]
ZnS:Cu,Co (510 nm)	TBrRh123 (518 nm)	120 keV, 2 Gy	[72]
LiYF_4_:Ce (305 nm)	ZnO (290 nm)	220 keV, 8 Gy	[73]
LiGa_5_O_8_:Cr (720 nm)	NC ^3^ (775 nm)	50 keV, 5 Gy	[74]
NaLuF_4_:Gd,Eu (543 nm)	Rose Bengal (560 nm)	160 keV, 5 Gy	[75]
Y_2.99_Pr_0.01_Al_5_O_12_@SiO_2_ (300–450 nm)	PpIX ^1^ (408 nm)	1.48 keV	[76]

^1^ Protoporphyrin, ^2^ Meso-tetra(4-carboxyphenyl)porphyrin, ^3^ 2,3-naphthalocyanine.

**Table 3:** Summary of the contribution of different energy sources in overcoming the limitations of PDT.

Energy Source	Contributions to Enhance the PDT
Microwaves	↑ Penetration depth ↑ Blood flow
Infrared light	↑ Penetration depth ↓ Phototoxicity in healthy tissues
X-ray	↑ Penetration depth
Ultrasound	↑ Penetration depth ↑ Precision in energy delivery
Electric field	↑ Transmembrane transport of PS and intracellular accumulation
Magnetic field	↑ Directional accumulation of PS in tumor cells ↓ Phototoxicity in healthy tissues

## Data Availability

Not applicable.
